# A Computational Approach to Estimating Nondisjunction Frequency in *Saccharomyces cerevisiae*

**DOI:** 10.1534/g3.115.024380

**Published:** 2016-01-08

**Authors:** Daniel B. Chu, Sean M. Burgess

**Affiliations:** Department of Molecular and Cellular Biology, University of California, Davis, California 95616

**Keywords:** meiosis, nondisjunction, precocious sister chromatid separation, budding yeast, tetrad analysis

## Abstract

Errors segregating homologous chromosomes during meiosis result in aneuploid gametes and are the largest contributing factor to birth defects and spontaneous abortions in humans. *Saccharomyces cerevisiae* has long served as a model organism for studying the gene network supporting normal chromosome segregation. Measuring homolog nondisjunction frequencies is laborious, and involves dissecting thousands of tetrads to detect missegregation of individually marked chromosomes. Here we describe a computational method (TetFit) to estimate the relative contributions of meiosis I nondisjunction and random-spore death to spore inviability in wild type and mutant strains. These values are based on finding the best-fit distribution of 4, 3, 2, 1, and 0 viable-spore tetrads to an observed distribution. Using TetFit, we found that meiosis I nondisjunction is an intrinsic component of spore inviability in wild-type strains. We show proof-of-principle that the calculated average meiosis I nondisjunction frequency determined by TetFit closely matches empirically determined values in mutant strains. Using these published data sets, TetFit uncovered two classes of mutants: Class A mutants skew toward increased nondisjunction death, and include those with known defects in establishing pairing, recombination, and/or synapsis of homologous chromosomes. Class B mutants skew toward random spore death, and include those with defects in sister-chromatid cohesion and centromere function. Epistasis analysis using TetFit is facilitated by the low numbers of tetrads (as few as 200) required to compare the contributions to spore death in different mutant backgrounds. TetFit analysis does not require any special strain construction, and can be applied to previously observed tetrad distributions.

Meiosis is an integral developmental program required for sexual reproduction in eukaryotes (Petronczki *et al.* 2003). Through two rounds of chromosome segregation, the DNA content of parent diploid cells (2*n*) is reduced to form haploid gametes (1*n*). Homologous chromosomes separate from one another in the first meiotic division that follows meiosis I prophase (Figure 1). Proper separation requires a series of dynamic chromosome events that physically tether homologous chromosomes together to form a bivalent. Errors in chromosome segregation are the leading genetic cause of birth defects in humans (Hassold and Hunt 2001). Many instances of nondisjunction (ND) can be traced to defects occurring during meiosis I prophase. Failure to properly separate homologs during meiosis I anaphase can result in chromosome aneuploidy in the fertilized zygote, often resulting in miscarriage or still birth (Figure 1; Hassold and Hunt 2001). Notable examples of viable aneuploidy are trisomy 21, which is the cause of Down syndrome, and XXY, which is the cause of Klinefelter syndrome.

**Figure 1:**
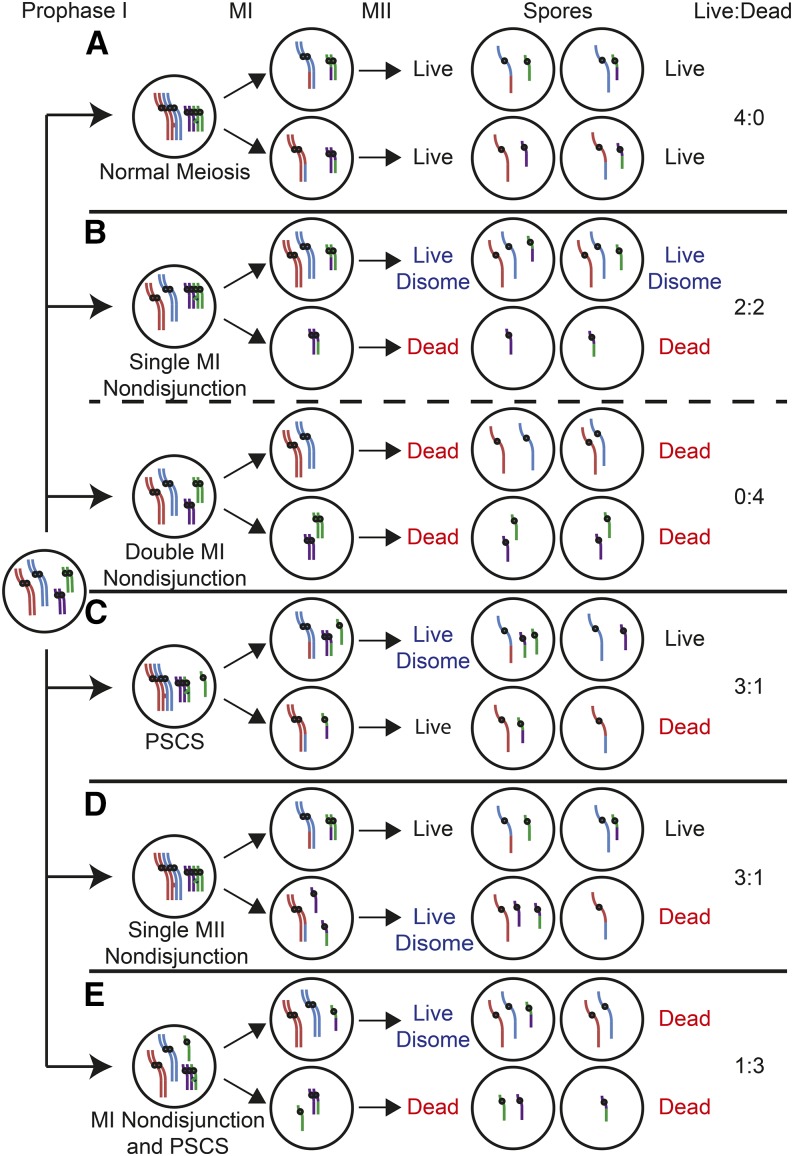
Schematic of different forms of nondisjunction. Newly replicated chromosomes (right) proceed to two sequential rounds of chromosome separation. The cell has two pairs of homologs, a long pair (red and blue), and a short pair (green and purple). Each homolog contains two sister chromatids represented by single lines. Every chromosome is essential, and the absence of either a long or short chromosome results in a dead spore. A spore with a single long and short chromosome is considered live and normal. A spore with either two long chromosomes and one short chromosome, or one long chromosome and two short chromosomes is considered a live disomes. The live disomes have a high probability of living but may die at a low frequency due to aneuploidy induced death (ANID, see *Materials and Methods*). (A, Normal meiosis) The long and short homologous chromosomes each undergo crossing over to ensure their proper segregation at MI. Following MII, each spore receives a long and a short chromosome resulting in four live spores. (B, Top; single MI nondisjunction) The long pair of chromosomes fails to form a crossover, resulting in an MI nondisjunction event where both chromosomes segregate to the same pole. Following MII, two spores are disomic for the long chromosome, and, in most cases produce live spores. The two spores missing a copy of the long chromosome die. (Bottom; MI double-nondisjunction) Both the long and short chromosomes fail to form a crossover, leading to a double nondisjunction where each pair of homologs separate to opposite poles. The result is four dead spores with two spores missing a long chromosome, and two spores missing a short chromosome. (C, Precocious sister chromatid separation) A loss of sister chromatid cohesion on a pair of short sister chromatids causes a sister chromatid to fail to properly disjoin during MI segregation. The sister chromatid segregates randomly during MI segregation, resulting in two live spores, one live but disomic spore with an extra short chromosome, and one dead spore missing a short chromosome. (D, Single MII nondisjunction) Meiotic prophase and MI segregation are normal. Before MII segregation there is loss of sister chromatid cohesion on a pair of short sister chromatids causing them to segregate to the same pole. There are two live spores, one live but disomic spore with an extra short chromosome, and one dead spore missing a short chromosome. (E, MI nondisjunction and precocious sister chromatid separation) The long pair of chromosomes fail to form a crossover, and there is a loss of sister chromatid cohesion between a pair of short sister chromatids. Only one spore is live but disomes and the rest of the spores are dead because they are missing either a long or short chromosome.

In female mammals, meiosis I prophase occurs in ovaries of the fetus, yet cells are blocked from completing anaphase until sexual maturity (Nagaoka *et al.* 2012). Thus, these contacts must be robust enough to last through decades spanning a reproductive lifespan. A maternal age effect increases the incidence of meiosis I (MI) chromosome missegregation in these gametes with compromised homolog attachments. The increased incidence of miscarriages in older women is likely due to the increased levels of MI nondisjunction (MI-ND; Hassold and Hunt 2001; Nagaoka *et al.* 2012).

Budding yeast has long served as an excellent model organism for studying the chromosome events of MI prophase. Forward genetic screens have identified dozens of conserved genes involved in key events such as homolog pairing, crossing over by homologous recombination, rapid chromosome motion, and the formation of the monopolar attachment of sister chromatid pairs, which ensures proper homolog disjunction (Esposito *et al.* 1970; Roeder 1995). The ability to characterize these events in mutant strains is facilitated by the ability to separate the four haploid spore products of meiosis from a single tetrad.

Mutants defective for processes related to the pairing, recombination, or synapsis of homologous chromosomes show decreased levels of spore viability due to MI-ND (Figure 1). While the presence of an extra chromosome (*n* + 1) generally supports viability, the loss of any one chromosome will result in spore death (Campbell and Doolittle 1987; Torres *et al.* 2007; St Charles *et al.* 2010). Thus, the outcome of a single MI-ND event generally results in a tetrad containing two live disomic spores, and two nullosomic dead spores (Figure 1B; Ross-Macdonald and Roeder 1994; Sym and Roeder 1994). Two MI-ND events will result in either a 0:4 live:dead tetrad if the homolog pairs segregate away from each other, or a 2:2 live:dead tetrad if the two homolog pairs segregate to the same pole.

Other types of meiotic errors causing spore inviability will appear as random spore death (RSD), which is the frequency any given spore will die, and that the viability of that spore is independent of the viability of any other spore. One example of RSD would be the nondisjunction of a single sister chromatid due to precocious sister chromatid separation (PSCS) or meiosis II nondisjunction (MII-ND); either event would result in a 3:1 live:dead tetrad (Figure 1, C and D). Inviable spores could also arise in cases unrelated to chromosome ND. These include defects in prospore membrane or spore wall formation, improper partitioning of organelles or other essential cytoplasmic components, or when defects in essential cellular processes lead to germination defects (Neiman 2011). In addition, defects in meiotic double-strand break repair can result in lethal lesions independent of segregation defects (Hochwagen and Amon 2006). Combinations of MI-ND and RSD can result tetrads containing 3, 2, 1, or 0 viable spores (Figure 1, B and E).

In previous studies, a high incidence of 2:2 and 0:4 tetrads has been interpreted as one indicator of increased MI-ND (*e.g.*, in mutants showing decreased levels of meiotic recombination; Ross-Macdonald and Roeder 1994; Sym and Roeder 1994). A quantitative description of this qualitative observation is lacking, and there is little mechanistic insight that can be made from this observation alone. For example, one shortcoming of relying on these events as an indicator of MI-ND is that increases in MI-ND may be difficult to observe in some strain backgrounds if significant, independent contributions to spore death are also at play (above).

Confirmation of increased MI-ND can be made by analyzing MI-ND frequencies of individual chromosomes (see references in Table 1). These measurements are typically carried out on a chromosome-by-chromosome basis, and can require dissecting up to thousands of tetrads to generate accurate MI-ND frequencies. Such assays typically involve the detection of segregating heterozygous codominant genetic markers among the viable spore clones. Examples of this approach include cosegregation of *MATa*/*MATα* to form nonmating spore clones for chromosome III, and the use of *CEN*-linked genetic markers (*e.g.*, *URA3*/*TRP1)* for other chromosomes (Wanat *et al.* 2008). Alternatively, fluorescent methods can be used to detect MI-ND in intact tetrads. One method is to tag specific chromosomes with large repetitive arrays of *tetO* or *lacO* DNA sequence (Straight *et al.* 1996; Michaelis *et al.* 1997; Marston *et al.* 2004). The chromosome can then be tracked by expressing either TetR or LacI fused to a fluorophore, [*e.g.*, green fluorescent protein (GFP)], which will bind to the arrays, allowing the presence of the tagged chromosomes to be visualized. Alternatively, a homolog pair can be engineered to express two different fluorophores from each homolog (Thacker *et al.* 2011). In the case of MI-ND, some spores will harbor multiple fluorophores, while others will have none. Both fluorescence methods have the advantage that many tetrads can be assayed without dissection; however, substantial strain construction carrying all relevant markers is first required.

**Table 1:** Measured MI-ND frequencies and computationally generated Avg-ND frequencies

Genotype	Chromosome	Method	MI-ND	Source
WT	I	*CEN1*	1.5%	(Cheslock *et al.* 2005)
WT	III	*MAT*	<0.1%	(Zanders and Alani 2009)
WT	III	*CEN3*	<0.1%	(Conrad *et al.* 1997)
WT	III	*CEN3*	<0.1%	(Chua and Roeder 1997)
WT	III	*CEN3*	<0.1%	(Chua and Roeder 1997)
WT	III	*MAT*	<0.1%	(Wang *et al.* 1999)
WT	III	*CEN3*	<0.1%	(Lee *et al.* 2012)
WT	III	*MAT*	<0.5%	(Hunter and Borts 1997)
WT	III	*lacO* array	1%	(Shonn *et al.* 2000)
WT	IV	*lacO* array	1.5%	(Shonn *et al.* 2000)
WT	V	*tetO* array	<0.5%	(Marston *et al.* 2004)
WT	VII	*lacO* array	1.5%	(Shonn *et al.* 2000)
WT	VIII	*lacO* array	1%	(Shonn *et al.* 2000)
WT	VIII	Fluorescent spore	<0.1%	(Thacker *et al.* 2011)
WT	Avg-ND	TetFit	0.3%	(Shonn *et al.* 2000)
WT	Avg-ND	TetFit	0.6%	(Martini *et al.* 2006)
WT	Avg-ND	TetFit	0.3%	(Wanat *et al.* 2008)
WT	Avg-ND	TetFit	0.5%	(Keelagher *et al.* 2011)
WT	Avg-ND	TetFit	0.2%	(Ghosh *et al.* 2004)
WT	Avg-ND	TetFit	0.4%	(Masison and Baker 1992)
WT	Avg-ND	TetFit	0.1%	(Jessop *et al.* 2006)
WT	Avg-ND	TetFit	0.1%	(Jessop *et al.* 2006)
WT	Total Avg-ND		0.3%	
*mad1*	III	*CEN3*	4.5%	(Cheslock *et al.* 2005)
*mad1*	III	*TetO* array	6%	(Marston *et al.* 2004)
*mad1*	Avg-ND	TetFit	7.4%	(Shonn *et al.* 2000)
*mad2*	I	*CEN1*	6%	(Cheslock *et al.* 2005)
*mad2*	III	*lacO* array	2%	(Shonn *et al.* 2000)
*mad2*	III	*tetO* array	15%	(Marston *et al.* 2004)
*mad2*	IV	*lacO* array	16%	(Lacefield and Murray 2007)
*mad2*	IV	*lacO* array	18%	(Shonn *et al.* 2000)
*mad2*	VII	*lacO* array	15%	(Shonn *et al.* 2000)
*mad2*	VIII	*lacO* array	11%	(Shonn *et al.* 2000)
*mad2*	Avg-ND	TetFit	6.5%	(Shonn *et al.* 2000)
*spo11-HA*	III	*MAT*	<0.1%	(Zanders and Alani 2009)
*spo11-HA*	VIII	Fluorescent spore	<0.15%	(Thacker *et al.* 2011)
*spo11-HA*	Avg-ND	TetFit	0.7%	(Martini *et al.* 2006)
*spo11-yf*	VIII	Fluorescent spore	1.7%	(Thacker *et al.* 2011)
*spo11-yf*	Avg-ND	TetFit	3.5%	(Martini *et al.* 2006)
*csm4*	III	*CEN3*	1.9%	(Lee *et al.* 2012)
*csm4*	III	*MAT*	7.8%	(Wanat *et al.* 2008)
*csm4*	III	*tetO* array	12%	(Marston *et al.* 2004)
*csm4*	XV	*CEN15*	1.4%	(Wanat *et al.* 2008)
*csm4*	Avg-ND	TetFit	5.5%	(Wanat *et al.* 2008)
*ndj1*	III	*CEN3*	0.3%	(Chua and Roeder 1997)
*ndj1*	III	*CEN3*	1.2%	(Chua and Roeder 1997)
*ndj1*	III	*CEN3*	1.7%	(Lee *et al.* 2012)
*ndj1*	III	*CEN3*	1.8%	(Conrad *et al.* 1997)
*ndj1*	III	*tetO* array	6%	(Marston *et al.* 2004)
*ndj1*	Avg-ND	TetFit	3.5%	(Wanat *et al.* 2008)
*msh5*	III	*MAT*	7.1%	(Wanat *et al.* 2008)
*msh5*	III	*tetO* array	10%	(Marston *et al.* 2004)
*msh5*	III	*MAT*	15.3%	(Hollingsworth *et al.* 1995)
*msh5*	VIII	Fluorescent spore	10.8%	(Thacker *et al.* 2011)
*msh5*	VIII	Fluorescent spore	12.7%	(Thacker *et al.* 2011)
*msh5*	XV	*CEN15*	3.4%	(Wanat *et al.* 2008)
*msh5*	Avg-ND	TetFit	12.0%	(Wanat *et al.* 2008)
*mlh1*	I	CHEF gel	7%	(Hunter and Borts 1997)
*mlh1*	III	*MAT*	1.5%	(Wang *et al.* 1999)
*mlh1*	III	*MAT*	6%	(Hunter and Borts 1997)
*mlh1*	III	*tetO* array	18%	(Marston *et al.* 2004)
*mlh1*	X	CHEF gel	5%	(Hunter and Borts 1997)
*mlh1*	XV	*CEN15*	1.2%	(Wanat *et al.* 2008)
*mlh1*	10 Chr	CHEF gel	3.8%	(Hoffmann *et al.* 2003)
*mlh1*	Avg-ND	TetFit	5.1%	(Wanat *et al.* 2008)
*exo1*	III	*MAT*	2.8%	(Keelagher *et al.* 2011)
*exo1*	III	*MAT*	11.8%	(Kirkpatrick *et al.* 2000)
*exo1*	III	*tetO* array	16%	(Marston *et al.* 2004)
*exo1*	XV	*CEN15*	4.2%	(Kirkpatrick *et al.* 2000)
*exo1*	Avg-ND	TetFit	6.4%	(Keelagher *et al.* 2011)
*sgs1*	III	*tetO* array	13%	(Marston *et al.* 2004)
*sgs1*	IV	RSA, *CEN4*	2.2%	(Watt *et al.* 1995)
*sgs1*	Avg-ND	TetFit	0.5%	(Jessop *et al.* 2006)
*cbf1*	III	*tetO* array	22%	(Marston *et al.* 2004)
*cbf1*	Chr fragment	Nutrition markers	7.8%	(Masison and Baker 1992)
*cbf1*	Avg-ND	TetFit	2.2%	(Masison and Baker 1992)
*iml3*	III	*tetO* array	17%	(Marston *et al.* 2004)
*iml3*	V	*tetO* array	6.0%	(Fernius and Marston 2009)
*iml3*	V	*tetO* array	10.5%	(Marston *et al.* 2004)
*iml3*	Avg-ND	TetFit	3.4%	(Ghosh *et al.* 2004)

The best-fit Avg-ND values generated by TetFit are listed under MI-ND using the live:dead tetrad distributions listed under source. Total Avg-ND is an average of all of the WT Avg-NDs. MI, meiosis I; ND nondisjunction.

A chromosome-by-chromosome approach also has limitations. First, if all chromosomes were to have an equal chance of undergoing MI-ND, and only 1 out of 16 chromosomes is analyzed, then only ∼6% of an already rare event can be detected. Accordingly, several thousands of tetrads generally need to be dissected to detect a few rare MI-ND events for only a subset of chromosomes. Second, MI-ND frequencies will be underestimated in mutants where there are high levels of 0:4 live:dead tetrads. Third, it is possible that MI-ND is nonrandomly distributed across the 16 chromosomes for any number of reasons (*e.g.*, chromosome size effect). For example, the *mad2*Δ mutation, which abolishes the spindle-assembly checkpoint, causes large chromosomes to nondisjoin at a higher frequency than small chromosomes (Shonn *et al.* 2000). Therefore, unless three or more chromosomes of different sizes are assayed, any negative results could be misleading.

Here, we offer a computational method (TetFit) to quantify the independent contributions of MI-ND and RSD to spore inviability based on finding the best-fit distribution of 4:0, 3:1, 2:2, 1:3, and 0:4 live:dead spores to an observed distribution for any given genotype. This holistic approach overcomes some of the limitations of measuring MI-ND frequencies of individual chromosomes. TetFit can be applied to any set of tetrad data to determine a calculated average MI-ND frequency (Avg-ND) for any given strain, and even other yeast species. Data mining of published data sets can also uncover heretofore unknown contributions to the fidelity of MI chromosome segregation. We validated TetFit using published data of WT, and mutant strains of budding yeast sets, and uncovered two classes of mutants that skew to either to MI-ND or RSD as the main contributors to spore inviability. We also show that as few as 200 tetrads can be dissected to distinguish MI-ND frequencies for two related mutants.

## Materials and Methods

### Calculating the distribution of tetrad types based on random spore death

TetSim is an R-script that simulates the expected distribution of tetrads giving 4, 3, 2, 1, or 0 viable spores due to RSD (Supporting Information, File S1). Here we define RSD as the frequency any given spore will die and that the viability of that spore is independent of the viability of any other spore. We simulated the distributions rather than using a binomial expansion to calculate the distributions so that we could account for data sets with low numbers of tetrads. Moreover, it allowed us to visualize how observed live:dead tetrad frequencies differed from the simulated distributions. In brief, the TetSim generates a matrix with the dimensions of 4 × number of tetrads set by the user that is then filled with computer-generated random fractional numbers of a uniform distribution between 0 and 1. The random numbers are then converted to 1 (live), if the number is less than the spore viability set by the user (*e.g.*, the random number 0.7 will be converted to 1 if the spore viability is 0.85), or 0 (dead), if the number is greater than the spore viability set by the user. Each set of four spores generated are then converted to a hypothetical tetrad giving 4, 3, 2, 1, or 0 viable spores, and the frequency of each tetrad class is calculated and recorded. This process is then repeated for the specified number of simulations, and the distributions can be plotted using the Beeswarm package of R. For this paper, 50,000 simulations were recorded each time TetSim was used.

### Modeling the effects of RSD and MI-ND on live:dead tetrad distributions

We created TetFit (File S2), an R-Script, which generates a matrix of live:dead tetrad distributions representing possible pairwise combinations of RSD and MI-ND frequencies using the user-set ranges (the defaults are 0 < RSD < 0.8 and 0 < MI-ND < 0.017) and the user-set intervals (the defaults are 500 for both RSD and MI-ND). The computationally-generated live:dead tetrad frequencies are then compared to the observed live:dead tetrad frequencies to find the RSD and MI-ND frequencies that best fit the observed data, based on the highest *P*-value generated by a chi-squared test.

To generate the live:dead tetrad frequencies, TetFit first calculates the effects of each RSD generated above for each number of dead spores (*D*, 0–4). A binomial expansion is used to determine the frequency of each *D* solely from the contributions of RSD ($fDRSD_{D}$) using the equation:

$$fDRSD_{D}=\frac{4!}{D!\left(4-D\right)!}*{\left(1-RSD\right)}^{D\;}*RSD^{4-D}$$ (Equation 1)

TetFit next calculates the effects of MI-ND on live:dead tetrad frequencies. TetFit takes into account several features of chromosome aneuploidy. First, MI-ND is defined in the classical sense in which two homologs fail to properly disjoin and segregate to the same pole (Figure 1B). We assumed that every chromosome is essential. As most disomies are well tolerated in yeast, most tetrads that have undergone a single MI-ND event will give two live and two dead spores (Torres *et al.* 2007; St Charles *et al.* 2010).

**Figure 2:**
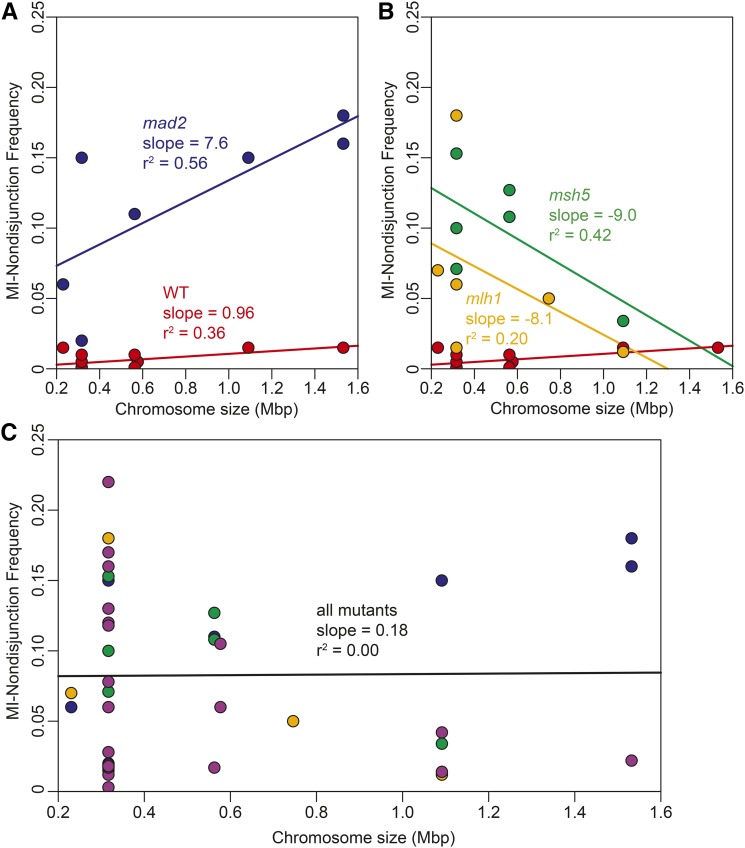
Chromosome size effect. Scatter plots of previously published MI-ND frequencies compared to chromosome size. References for all data points can be found in Table 1. (A) Scatter plot and best fit lines for WT (red) and *mad2*Δ (blue). (B) Scatter plot and best fit lines for WT (red), *msh5*Δ (green), and *mlh1*Δ (orange). (C) Scatter plot and best fit line for *mad2*Δ (blue), *msh5*Δ (green), *mlh1*Δ (orange), and all other mutant MI-ND data from Table 1, excluding spo11-HA, which does not have a MI-ND defect (purple). The best fit line was generated from all data points plotted. The chromosomes analyzed for MI-ND include the shortest yeast chromosome, Chr I (230 kbp), and the longest yeast chromosome, Chr IV (1532 kbp). Other yeast chromosomes analyzed include Chr III (317 kbp), Chr V (577 kbp), Chr VII (1091 kbp), Chr VIII (563 kbp), Chr X (746 kbp), and ChrXV (1091 kbp).

For this model, to simplify the calculations, we assumed that the MI-ND frequency for each chromosome is equal. Since we cannot predict *a priori* how any given chromosome may be affected in any given mutant, we assume the null hypothesis to be true. We compiled 10 different published studies where individual chromosomes from wild type (WT) strains were assayed for MI-ND frequency (Table 1). We found a very modest trend toward increased MI-ND with increasing chromosome size (Figure 2A). The *mad2*Δ mutant is the only example that we know of where a chromosome size effect has been measured for enough chromosomes to show a significant trend (Shonn *et al.* 2000). We expanded the results from Shonn *et al.* (2000) to include data from three other studies, and plotted MI-ND frequencies for individual chromosomes based on size (Table 1 and Figure 2, B and C). A trend line fit to the *mad2*Δ data showed a positive correlation (*r*^2^ = 0.56). Anecdotal evidence suggests that the opposite is true for mutants affecting meiotic recombination. For the *mlh1*Δ and *msh5*Δ mutants, the data showed a weak negative correlation (*r*^2^ = 0.20 and 0.42, respectively; Table 1 and Figure 2B). The slopes and correlations for both *mlh1*Δ and *msh5*Δ appeared to be dependent on a single outlier point (Figure 2B). We next combined all of the MI-ND data for all of the mutants we could find data for to determine if an overall mutant chromosome size effect trend could be detected (Table 1 and Figure 2C). There was no correlation between chromosome size and MI-ND in the combined data set (*r*^2^ = 0.00; Figure 2C).

The incidence of multiple MI-ND events do not follow a Poisson distribution (Shonn *et al.* 2000); that is, there are more multi-chromosome MI-ND events than expected from a random distribution. This is likely due to the distributive or achiasmate segregation mechanism that segregates nonexchange chromosomes to opposite poles (Kurdzo and Dawson 2015). This segregation is directed by homology independent pairing of centromeres (Kemp *et al.* 2004; Gladstone *et al.* 2009; Newnham *et al.* 2010). In the case of a single achiasmate homolog pair, this distributive mechanism can direct proper segregation; however, in the case of multiple achiasmate chromosomes, the distributive mechanism will be overwhelmed. To account for the nonrandom distribution of MI-ND events, we included an MI-ND multiplier to increase the number of multi-chromosome MI-ND events.

For each number of nondisjunction chromosomes (NC, 0–16), TetFit uses the MI-ND frequency (ND) to calculate the frequency of nondisjunction for NC chromosomes per meiosis ($f{\text{NDJ}}_{\text{NC}}$). We used of a binomial expansion to calculate $f{\text{NDJ}}_{\text{NC}}$ since it was a simple and logical starting place compared to other possible distributions (*e.g.*, a Poisson distribution). A weakness of the Poisson distribution is that the tail-end of the distribution will include nondisjunction chromosome values greater than the number of yeast chromosomes. The binomial distribution has the advantage that yeasts with fewer than 16 chromosomes (*e.g.*, *Schizosaccharomyces pombe*, *n* = 3) can be easily accommodated by the model, which would not apply to a Poisson distribution. Moreover, we go to great lengths in the paper to show that it models MI-ND quite well. As is, we feel the current solution is sufficient for its intended use. To calculate $f{\text{NDJ}}_{\text{NC}}$ TetFit uses the equation:

$$f{\text{NDJ}}_{\text{NC}}=\frac{16!}{\text{NC}!\left(16-\text{NC}\right)!}*{\left(1-\text{ND}\right)}^{16-\text{NC}\;}*{\text{ND}}^{\text{NC}}$$ (Equation 2)

When NC > 1, ND is multiplied by the MI-ND multiplier to account for the nonrandom distribution of MI-ND events. An empirically determined MI-ND multiplier of 10 gave good best-fit live:dead tetrad distributions for the combined Shonn, Martini, and Wanat data sets as a test case.

Without the MI-ND multiplier, Equation 2 will generate a probability distribution under all conditions. Equation 2 will also calculate a probability distribution that sums to 1 when the MI-ND multiplier is set at the default (10), and MI-ND is less than 0.017. Any increase in the frequency of MI-ND due to the MI-ND multiplier is offset by a decrease in the frequency of cells with no MI-ND. Under the default parameters, all of the individual frequencies will be positive and sum to 1. TetFit will generate negative frequencies when the increase from the MI-ND multiplier is greater than the frequency of cells with no MI-ND; this will only occur under extremely high levels of MI-ND (*e.g.*, when spore inviability due to MI-ND > 75%). Using the default settings will prevent this.

In the case of multiple MI-ND events (NC > 1), homolog pairs segregate randomly to each pole. The fraction of cells for which all nondisjunction homolog pairs segregate to a single pole for each NC ($f{\text{SP}}_{\text{NC}}$*)* is given by the equation:

$$f{\text{SP}}_{\text{NC}}=0.5^{\text{NC}-1}$$ (Equation 3)

Though disomy is generally tolerated and viable, disomic strains have growth defects and reduced germination rates (Campbell and Doolittle 1987; Torres *et al.* 2007; St Charles *et al.* 2010). The effects of multiple disomies appear to be relatively low since spore viability from triploid meiosis, where nearly every spore would contain multiple disomic chromosomes, can be as high as 50–83% (Campbell and Doolittle 1987; St Charles *et al.* 2010). To incorporate the negative effects of an additional chromosome, we added concept of aneuploidy-induced death for a single chromosome aneuploidy (ANID). To account for the negative effects of multiple aneuploidies, we use a model where aneuploidy-induced death increases with the number of nondisjunction chromosomes (${\text{ANID}}_{\text{NC}}$).

We empirically determined that an ANID of 0.035 was able to produce good fits for the Shonn, Martini, and Wanat data sets. To calculate ${\text{ANID}}_{\text{NC}}$ TetFit uses the equation:

$${\text{ANID}}_{\text{NC}}=1-{\left(1-\text{ANID}\right)}^{\text{NC}}$$ (Equation 4)

We assumed that effects of aneuploidy on the two surviving spores were independent, such that the viability or inviability of one spore did not affect the other spore. TetFit calculates the frequency of aneuploidy-induced death of 0, 1, and 2 spores per meiosis ($f{\text{ANID0}}_{\text{NC}}$, $f{\text{ANID1}}_{\text{NC}}$, and $f{\text{ANID2}}_{\text{NC}}$, respectively) for each NC using the following equations:

$$f{\text{ANID0}}_{\text{NC}}={\left(1-{\text{ANID}}_{\text{NC}}\right)}^{2}$$ (Equation 5)

$$f{\text{ANID1}}_{\text{NC}}=2*{\text{ANID}}_{\text{NC}}*(1-{\text{ANID}}_{\text{NC}})$$ (Equation 6)

$$f{\text{ANID2}}_{\text{NC}}={\text{ANID}}_{\text{NC}}{}^{2}$$ (Equation 7)

TetFit calculates the frequency of 2, 3, or 4 spores dead ($f2{\text{SD}}_{\text{NC}}$, *$f{\text{3SD}}_{\text{NC}}$*, and $f4{\text{SD}}_{\text{NC}}$, respectively) due to MI-ND from NC nondisjunction chromosomes, and ANID using the following equations:

$$f2{\text{SD}}_{\text{NC}}=f{\text{NDJ}}_{\text{NC}}*f{\text{SP}}_{\text{NC}}\;*\;f{\text{ANID0}}_{\text{NC}}$$ (Equation 8)

$$f3{\text{SD}}_{\text{NC}}=f{\text{NDJ}}_{\text{NC}}*f{\text{SP}}_{\text{NC}}\;*\;f{\text{ANID1}}_{\text{NC}}$$ (Equation 9)

$$f4{\text{SD}}_{\text{NC}}=f{\text{NDJ}}_{\text{NC}}*f{\text{SP}}_{\text{NC}}*\;f{\text{ANID2}}_{\text{NC}}+f{\text{NDJ}}_{\text{NC}}*(1-f{\text{SP}}_{\text{NC}})$$ (Equation 10)

TetFit next calculates the total frequencies of 2, 3, or 4 spores dead from all MI-ND events (f2SD, *f3SD*, and *f4SD*) by summing all of the $f2{\text{SD}}_{\text{NC}}$, $f3{\text{SD}}_{\text{NC}}$, and $f4{\text{SD}}_{\text{NC}}$, respectively, using the following equations:

$$f2\text{SD}=\overset{16}{\underset{\text{NC}=1}{\sum }}f2{\text{SD}}_{\text{NC}}$$ (Equation 11)

$$f3\text{SD}=\overset{16}{\underset{\text{NC}=1}{\sum }}f3{\text{SD}}_{\text{NC}}$$ (Equation 12)

$$f4\text{SD}=\overset{16}{\underset{\text{NC}=1}{\sum }}f4{\text{SD}}_{\text{NC}}$$ (Equation 13)

Finally, to calculate the final frequencies of each live:dead tetrad class, we used the tetrad distributions generated from the RSD modeled distribution, and then applied the spore death due to MI-ND to find the final frequencies of tetrads with 4, 3, 2, 1, and 0 live spores (f4L, f3L, f2L, f1L, and f0L, respectively). We assumed that inviability due to MI-ND and RSD occur completely independently of each other, such that a spore dying from MI-ND does not affect its chance of dying from RSD. Moreover, a spore may be “killed twice” by both death from MI-ND and RSD. Equations 14–18 were derived as follows:

First, the frequencies of 4:0 and 3:1 tetrads are simply the fraction of tetrads with 0 or 1 spores dying due solely to RSD ($f{\text{DRSD}}_{0}$ and $f{\text{DRSD}}_{1}$ from Equation 1), respectively.

$$f4\text{L}=f{\text{DRSD}}_{0}*(1-f2\text{SD}-f3\text{SD}-f4\text{SD})$$ (Equation 14)

$$f3\text{L}=f{\text{DRSD}}_{1}*(1-f2\text{SD}-f3\text{SD}-f4\text{SD})$$ (Equation 15)

Second, to calculate the final frequency of 2:2 live:dead tetrads (f2L) we considered all combinations of RSD and MI-ND that could give this distribution. For the simplest cases, 2:2 tetrads can arise when two spores die due to only to RSD $\left(f{\text{DRSD}}_{2}\right)$, or when two spores die due to only a single, MI-ND event $\left(f{\text{DRSD}}_{0}*f2\text{SD}\right)$. Another contributing class includes the tetrads where the two dead spores have been killed twice by RSD and MI-ND. This frequency, 1/6, is analogous to the one out of six chances that the segregation of two unlinked genetic markers results in a nonparental ditype (Sherman 1991). For the fraction of tetrads that had either 0 or 1 spore die due to RSD, the inclusion of an MI-ND event will result in a 2:2 live:dead tetrad one-half of the time$\;\left(\frac{1}{2}f{\text{DRSD}}_{1}*f2\text{SD}\right),$ while the other half will give 1:3 live:dead tetrads (see Equation 17). Thus, TetFit calculates the final frequency of 2:2 live:dead tetrads using Equation 16:

$$f2\text{L}=f{\text{DRSD}}_{2}*\left(1-\frac{5}{6}f2\text{SD}-f4\text{SD}-f3\text{SD}\right)+(f{\text{DRSD}}_{0}*f2\text{SD})+(\frac{1}{2}f{\text{DRSD}}_{1}*f2\text{SD})$$ (Equation 16)

We used a similar logic to calculate the final frequency of 1:3 live:dead tetrads (f1L) by considering the fraction of three spores dying from RSD alone $(f{\text{DRSD}}_{3}$) and the combinations of RSD and MI-ND events using Equation 17.

$$f1\text{L}=f{\text{DRSD}}_{3}*\left(1-\frac{1}{2}f2\text{SD}-\frac{3}{4}f3\text{SD}-f4\text{SD}\right)+(f{\text{DRSD}}_{0}*f3\text{SD})+(\frac{1}{2}f{\text{DRSD}}_{1}*f2\text{SD})+(\frac{3}{4}f{\text{DRSD}}_{1}*f3\text{SD})+(\frac{2}{3}f{\text{DRSD}}_{2}*f2\text{SD})+(\frac{1}{2}f{\text{DRSD}}_{3}*f2\text{SD})$$ (Equation 17)

To calculate the final frequency of tetrads with 0 live spores, TetFit subtracts the frequency of 4, 3, 2, and 1 live spore tetrads using the following equation:

f0L=1−f4L−f3L−f2L−f1L (Equation 18)

To calculate average frequency of MI-ND per chromosome per cell (Avg−ND), TetFit uses the equation:

$$\text{Avg}-\text{ND}=\frac{1}{16}\overset{16}{\underset{\text{NC}=1}{\sum }}\text{NC}*f{\text{NDJ}}_{\text{NC}}$$ (Equation 19)

To calculate fraction of spores that are inviable from MI-ND due to MI-ND death (NDD) TetFit uses the equation:

$$\text{NDD}=\;\frac{1}{2}f2\text{SD}+\;\frac{3}{4}f3\text{SD}+\;f4\text{SD}$$ (Equation 20)

Although RSD and NDD can be directly compared to assess their relative contributions to spore inviability, they cannot directly be added together to determine spore viability because of RSD and NDD double-counted dead tetrads.

### Determining the best fit RSD and MI-ND

Once all of the live:dead tetrad distributions are calculated for each RSD and ND combination, each computationally generated live:dead tetrad distribution is compared to the observed distributions for each genotype using a chi-squared test. The live:dead tetrad distribution with the lowest chi-squared statistic is recorded as the best fitting tetrad for each genotype. When performing multiple comparisons, *P* values were adjusted using the Holm method (Holm 1979).

## Results

### RSD alone does not account for the observed distribution of live:dead tetrads from WT strains

**Figure 3:**
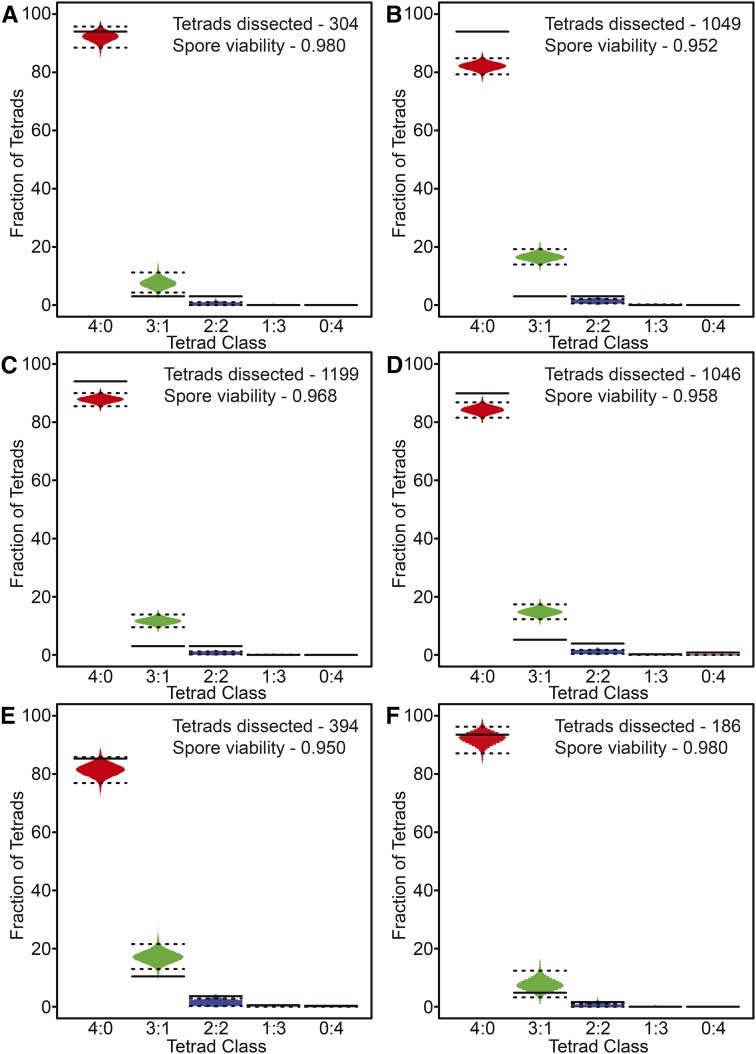
Simulation of random spore death in WT strains. Representative simulation of tetrad distributions using TetSim are based on the observed spore viability and number of tetrads dissected for the indicated WT data sets. A distribution of dissected tetrad simulations is shown [in gray (4:0), green (3:1), dark blue (2:2), light blue (1:3), and purple (0:4)] with the upper and lower dashed lines represent 99% and 1% percentiles, respectively. The observed frequencies of live:dead tetrad distributions for individual strains are shown as solid lines. (A)–(F) Comparison of simulated tetrad distributions with WT data from previously published data sets: (A) Shonn *et al.* 2000; (B) Martini *et al.* 2006; (C) Wanat *et al.* 2008; (D) Keelagher *et al.* 2011; (E) Masison *et al.* 1992; and (F) Jessop *et al.* 2006.

WT strains of budding yeast typically give ∼98% spore viability among tetrads (Figure 3). Rockmill *et al.* (2006) showed that chromosome III disomic spores could be recovered as a consequence of PSCS events, which would appear as RSD in this analysis. We wondered whether the 2% spore inviability in WT cells could be attributed solely to RSD, or if other factors were at play. To test this, we created TetSim to model the expected distribution of live:dead tetrads due to RSD, which includes PSCS and MII-ND, based on empirically measured spore viability frequencies. These calculated distributions were then compared to a set of six empirically measured data from WT strains, as reported in six previously published papers (Masison and Baker 1992; Shonn *et al.* 2000; Jessop *et al.* 2006; Martini *et al.* 2006; Wanat *et al.* 2008; Keelagher *et al.* 2011).

If the spore inviability in the six WT strains was due solely to RSD, then the observed distribution of live:dead tetrads should resemble the output of TetSim. We found, however, that the observed distributions reported for all WT strains fell outside of the 1–99^th^ percentiles of simulated data for at least one of the live:dead tetrad classes (Figure 3). Specifically, the published data showed an overrepresentation of 2:2 live:dead tetrads, and underrepresentation of 3:1 live:dead tetrad distributions compared to the simulation (Figure 3). The observed pattern could not be modeled by simply increasing or decreasing the simulated spore viability. That is, increasing the simulated spore viability would consequently decrease the frequency of 2:2 live:dead tetrads. Conversely, decreasing the simulated spore viability increased the frequency of 3:1 live:dead tetrads. These results suggest that spore inviability in WT strains is not due solely to RSD or PSCS. Instead, the increased incidence of 2:2 live:dead tetrads observed for WT compared to the simulation suggests that spore death may (in part) be due to an intrinsic level of MI-ND.

### RSD does not account for spore inviability in mutants defective for meiotic chromosome segregation

Using the same approach as for WT (above), we selected mutants with known meiotic chromosome segregation defects and compared the observed distributions of 4, 3, 2, 1 and 0 viable spore tetrads to distributions based on RSD. For this analysis, we included *mad1*Δ and *mad2*Δ mutants that have a defective spindle-assembly checkpoint (Shonn *et al.* 2000), the hypomorphic *SPO11* mutants that reduce double-strand break levels (*spo11-yf* and *spo11-df*; Martini *et al.* 2006), *ndj1*Δ and *csm4*Δ mutants defective for telomere-led motion (Wanat *et al.* 2008), and *msh5*Δ, *mlh1*Δ, and *exo1*Δ mutants with defects in homologous recombination (Wanat *et al.* 2008; Keelagher *et al.* 2011). We also included in our analysis *cbf1*Δ and *iml3*Δ mutants, which have defective centromeres (Masison and Baker 1992; Ghosh *et al.* 2004), and *sgs1*Δ and *sgs1*Δ*795* mutants that display increased PSCS due to increased crossover formation near centromeres (Jessop *et al.* 2006; Rockmill *et al.* 2006).

**Figure 4:**
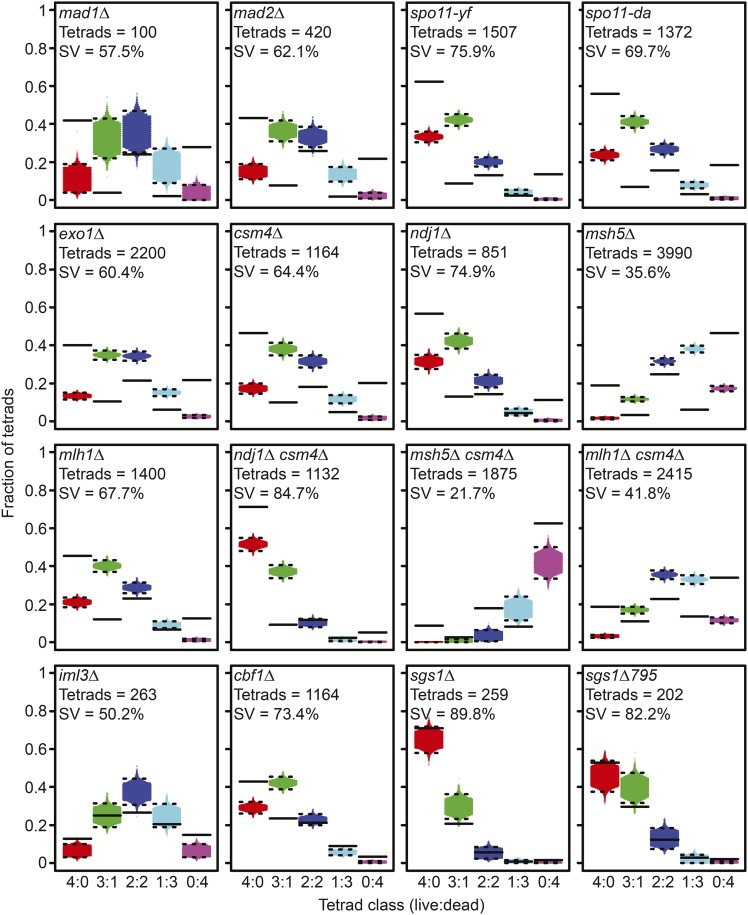
Simulation of random spore death in mutant strains. Representative simulation of tetrad distributions using TetSim based observed spore viability and number of tetrads dissected for the indicated mutant data sets. A distribution of dissected tetrad simulations is shown (in red, green, dark blue, light blue, and purple) with the upper and lower dashed lines represent 99% and 1% percentiles, respectively. The observed frequencies of live:dead tetrad distributions for individual strains are shown as solid lines. Comparison of simulated tetrad distributions with mutant data from previously published data sets generated by Shonn *et al.* (2000), Martini *et al.* (2006), Wanat *et al.* (2008), Keelagher *et al.* (2011), Masison *et al.* (1992), and Jessop *et al.* (2006).

All mutants gave one or more tetrad classes outside the 1 through 99^th^ percentiles based solely on RSD (Figure 4). These results point to factors other than RSD (or PSCS or MII-ND) contributing to spore death. Poor fits to the RSD model would be expected for mutants with increased MI-ND (Table 1). For *sgs1* mutants, where PSCS has been shown to contribute to spore inviability (Jessop *et al.* 2006; Rockmill *et al.* 2006), the poor fit to TetSim suggests that MI-ND may also contribute to spore inviability.

### Modeling MI-ND

We next tested if the differences in observed and expected RSD distributions for WT and mutant strains could be explained by MI-ND. For this we developed TetFit to model the relative contributions of RSD and MI-ND events that give the best fit to the observed data for WT and mutant strains. TetFit also generates the average MI-ND (Avg-ND) frequency, which is the frequency of any given chromosome undergoing a MI-ND event per meiosis.

The assumptions used to model MI-ND are described in detail in *Materials and Methods*. Briefly, MI-ND is defined in the classical sense in which the two homologs fail to properly disjoin and segregate to the same pole (Figure 1B). We assumed that every chromosome is essential, and that the MI-ND frequency for each chromosome is equal. As most disomies are well tolerated in yeast, most tetrads that have undergone a MI-ND event will give two live and two dead spores (Torres *et al.* 2007; St Charles *et al.* 2010). In the case of multiple MI-ND events, we assumed that the homologs would segregate randomly to each pole. We also accounted for the negative effect of multiple disomies on spore germination (Campbell and Doolittle 1987; Torres *et al.* 2007; St Charles *et al.* 2010). Finally, we considered that multi-chromosome MI-ND events occur at higher frequencies than expected from a Poisson distribution (Shonn *et al.* 2000).

### Modeled tetrad distributions closely match observed tetrad distributions in WT and mutant strains

**Figure 5:**
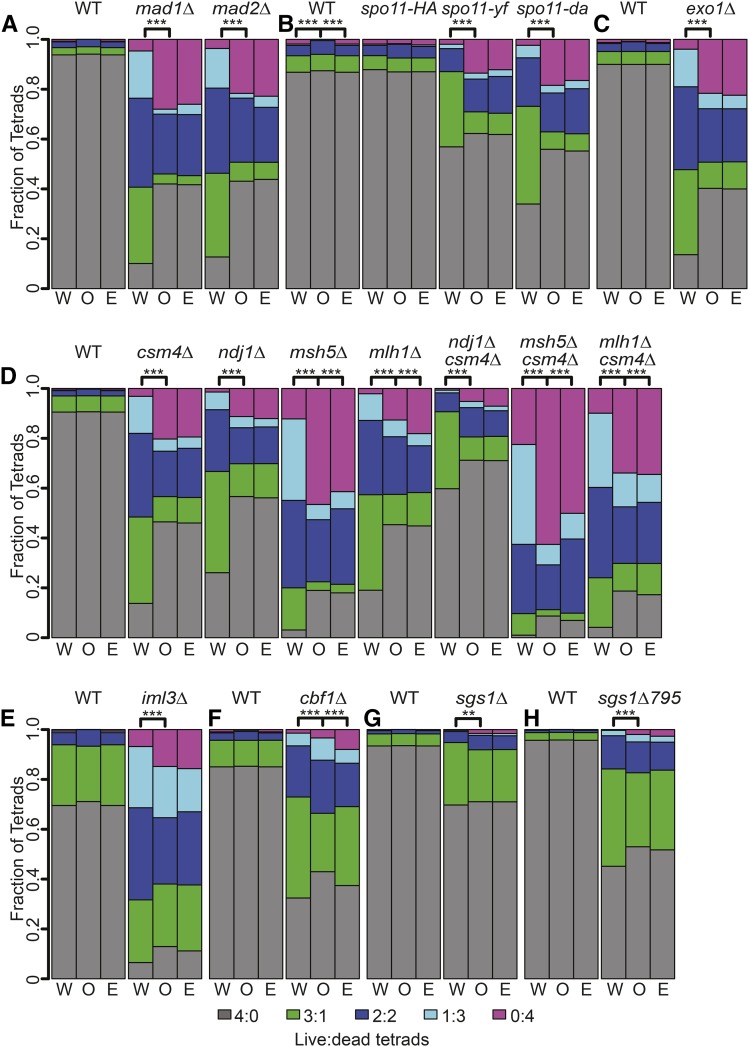
Comparison of observed and expected tetrad distributions. The observed tetrad distributions of genotype (O, middle bar), the expected tetrad distributions with the best fitting MI-ND and RSD (E, right bar), and the expected tetrad distributions using WT MI-ND and the best fitting RSD (W, left bar). Expected live:dead tetrad frequencies were generated for all genotypes using TetFit with the following conditions: the number of nondisjunction intervals (ndint) was set to 3000, the number of random spore death intervals (rsdint) was set to 3000, the number of chromosomes (chr) was set to 16, ANID was set to 0.035, and the MI-ND multiplier (ndm) was set to 10. (A) – (H) Comparison of simulated tetrad distributions with WT and mutant data from previously published data sets: (A) Shonn *et al.* (2000) data set; (B) Martini *et al.* (2006) data set; (C) Keelagher *et al.* (2011) data set; (D) Wanat *et al.* (2008) data set; (E) Ghosh *et al.* (2004) data set; (F) Masison *et al.* (1992) data set; (G) Jessop *et al.* (2006) data set; and (H) Jessop *et al.* (2006) data set. In these strains, the endogenous SGS1 has been deleted but *SGS1* or *sgs1*Δ*795* have been inserted at *TRP1* in the WT and *sgs1*Δ*795* strains, respectively. Significance is noted as ** *P* < 0.005; *** *P* < 0.0001 (chi-squared, Holm corrected).

TetFit-A finds the expected (E) RSD and MI-ND frequencies that give the best fit to the observed (O) tetrad distributions for each mutant (Figure 5). In 11 out of 15 strains described above, TetFit found combinations of MI-ND and RSD values giving best-fit distributions that were statistically indistinguishable from the observed distributions (Figure 5). These included mutants with known defects in chromosome segregation including *mad1*Δ, *mad2*Δ, *spo11-yf*, *spo11-da*, *exo1*Δ, *csm4*Δ, *ndj1*Δ, *csm4*Δ *ndj1*Δ, *iml3*Δ, *sgs1*Δ, and *sgs1-795*. For a subset of mutants, however, TetFit-A could not find a best-fit distribution. This result points to factors other than MI-ND and RSD that impact spore death in these strains. These cases are discussed in more detail below. In all cases where each mutant strain’s respective intrinsic WT MI-ND is used (TetFit-B), no distribution giving a good fit to the observed distribution could be generated. These findings are consistent with the interpretation that increased levels of MI-ND contribute to spore inviability in WT cells. The *spo11-HA* mutant was the only exception since the WT distribution was not significantly different; this would be expected since *spo11-HA* does not display a spore viability defect (Martini *et al.* 2006).

### Factors in addition to RSD and MI-ND contribute to spore inviability

While TetFit generated good fits for most mutants, exceptions were found for *cbf1*Δ, *mlh1*Δ, and *msh5*Δ. That is, no combination of MI-ND and RSD values could be generated to fit the observed distribution. Nonetheless, the distributions with the lowest *P*-values were qualitatively much more similar to the observed distribution than the distribution for RSD alone or with WT MI-ND values. Certainly, there could be other nonrandom factors contributing to spore inviability that are not accounted for by TetFit. We suggest that mutations or aneuploidies generated during mitotic growth (*cbf1*Δ, *mlh1*Δ) or poor sporulation efficiency (*msh5*Δ) could impart spore death independent of MI-ND or RSD.

Loss of *cbf1*Δ is known to increase nondisjunction during mitotic growth, which may cause a population of the dissected tetrads to be aneuploid (Baker and Masison 1990; Masison and Baker 1992). In the case of a single monosomy in diploid cells, there would be an increase the 2:2 live dead frequencies, because two spores would be nullosomic after meiosis. Similarly, the mutL homolog, Mlh1, plays a critical role in mismatch repair during mitotic growth, and loss of Mlh1 results in greatly increased mutation rates (Heck *et al.* 2006). Indeed, the cell divisions required for a single diploid *mlh1*Δ cell to form a colony can lead to the accumulation of large numbers of spontaneous recessive lethal mutations, leading to a sizeable loss of spore viability and increased incidence of 2:2 tetrads (Reenan and Kolodner 1992; Heck *et al.* 2006). The *mlh1*Δ dissection data we used was generated using a zero growth mating protocol, in which the two haploid strains are mated and then immediately induced to sporulate (Reenan and Kolodner 1992; Wanat *et al.* 2008). Despite this, it is likely that the haploids used to mate and sporulate would have accumulated nonlethal mutations during their initial growth. Some of these mutations would likely affect mitotic growth and germination, leading to the inability to form a visible colony. Moreover, the random segregation of mutations from each parent may result in synthetic lethal mutation combinations leading to spore inviability.

The *msh5*Δ mutation is not known to cause mitotic defects that could lead to altered live:dead tetrad frequencies. One possible explanation for the poor fit is that unrepaired meiotic recombination intermediates in these strains could introduce a variable that is not accounted for by TetFit. Another possibility of the poor fit for *msh5*Δ is that its low sporulation efficiency may lead to biases in the tetrad population dissected (Borner *et al.* 2004; Chan *et al.* 2009). The meiotic defects caused by *msh5*Δ are reduced at lower temperatures, leading to increased sporulation efficiency of *msh5*Δ cells at lower temperatures (Borner *et al.* 2004; Chan *et al.* 2009). The *msh5*Δ cells from the Wanat dataset were sporulated at 30°, a temperature at which sporulation efficiency is reduced compared to lower temperatures (Borner *et al.* 2004; Wanat *et al.* 2008; Chan *et al.* 2009). To test if decreased sporulation efficiency was the cause of the poor fits generated by TetFit, we examined a different dataset of *msh5*Δ sporulated at 23° or 33° with sporulation efficiencies of 68% and 21%, respectively (Chan *et al.* 2009). We found that TetFit generated better fitting distributions to the observed distributions for the 23° dissection than the 33° dissection (*P* = 0.105 and *P* = 0.039, respectively; Figure S1), indicating that a biased tetrad population may be the cause of TetFit poor fits to the *msh5*Δ strains.

These results do not necessarily detract from the utility of the computational method to identify mutants exhibiting increased MI-ND. Instead, these examples indicate that genotypes with high levels of spontaneous mutations or reduced sporulation efficiency can have altered spore inviability compared to that expected from RSD and MI-ND.

### Comparison of Avg-ND to observed MI-ND for individual chromosomes

The calculated Avg-ND for the WT strains described above ranged from 0.1% to 0.6% with an average of 0.3% (Table 1). These values are similar to empirically determined values for MI-ND frequencies for a limited set of individual chromosomes measured using different assays, which ranged from < 0.1–1.5% (Table 1). These results indicate that MI-ND contributes to spore inviability in WT strains.

Measurements of MI-ND frequencies in mutant strains can vary widely depending on the assay and chromosome analyzed (Table 1). For example, for the *mad2*Δ mutant, measurements using the same assay and the same strain background gave MI-ND frequencies ranging from 2% to 18% for different chromosomes suggesting a chromosome size effect, with longer chromosomes more likely to missegregate than shorter chromosomes (Table 1; Shonn *et al.* 2000). On the other hand, different assays measuring MI-ND of the same chromosome can be quite different. For example, Chr III measured in *mad2*Δ using the *lacO* or *tetO* array markers gave MI-ND frequencies of 2% and 15%, respectively (Table 1). Moreover, using CEN markers on Chr III in the *ndj1*Δ mutant gave MI-ND frequencies from 0.3% to 1.8%, while using a *tetO* array gave a MI-ND frequency of 6% (Table 1). TetFit calculates average MI-ND frequency for all chromosomes, thus eliminating the risk of picking a chromosome with much higher or lower level of MI-ND compared to other chromosomes. By this reasoning, we expected that the calculated Avg-ND frequency for WT and the mutants affecting MI-ND would fall within the range of observed frequencies for individual chromosomes.

For the mutants shown previously to increase MI-ND, the Avg-ND was within the range of measured values, if not slightly higher (Table 1). By contrast, for the mutants shown previously to increase PSCS, the calculated Avg-ND tended to be slightly lower (Table 1). This could be due to the fact that a subset of PSCS events may appear as MI-ND events in the given assays, which would not be included in the Avg-ND value. These results indicate that Avg-ND gives an approximate value of MI-ND, thus allowing direct comparisons between mutant strains.

### The predictive power of a computational approach to measuring nondisjunction frequency

**Figure 6:**
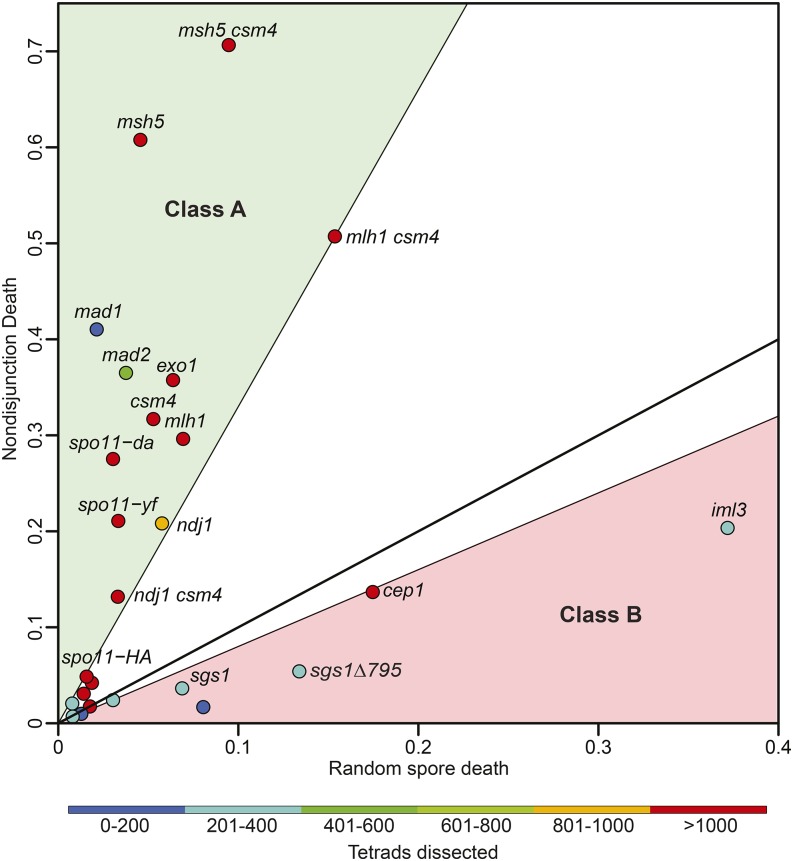
Comparison of random spore death and nondisjunction death. A scatter plot of the calculated best fits of RSD and NDD for all of the WT and mutant data sets analyzed. All WT strains are shown as unlabeled spots on the plot. Data points are colored based on the reported number of tetrads dissected. The solid line represents a NDD/RSD slope of 1, which indicates an equal contribution of NDD and RSD on spore inviability. The green shaded area covers the Class A mutants that have an NDD/RSD ratio above 3.3, and the pink shaded area covers Class B mutants that have an NDD/RSD ratio below 0.8.

MI-ND and RSD (including PSCS and MII-ND events) contribute independently to spore inviability (above). To directly compare the contributions of death from MI-ND and RSD, we computationally converted the expression of MI-ND frequency to MI-ND death (NDD). For WT strains, the contribution of NDD and RSD were roughly equal (Figure 6 and Table 2). Mutant strains appeared to fall into two classes: The first class (Class A) gave NDD/RSD > 3.3, while the second class (Class B) gave NDD/RSD < 0.8 (Figure 6). Not surprisingly, Class A genes include those with roles in homolog engagement and separation, while Class B genes include those with roles in sister chromatid cohesion and centromere function.

**Table 2:** Computationally derived random spore death and nondisjunction death

Genotype	RSD	NDD	NDD/RSD	Obs SV	Cal SV	Source
WT	0.008	0.021	2.657	0.978	0.972	(Shonn *et al.* 2000)
*mad1*	0.021	0.410	19.234	0.575	0.577	(Shonn *et al.* 2000)
*mad2*	0.038	0.365	9.708	0.621	0.611	(Shonn *et al.* 2000)
WT	0.019	0.042	2.255	0.952	0.940	(Martini *et al.* 2006)
*spo11-HA*	0.016	0.049	3.087	0.940	0.936	(Martini *et al.* 2006)
*spo11-yf*	0.033	0.211	6.323	0.759	0.763	(Martini *et al.* 2006)
*spo11-da*	0.030	0.275	9.055	0.697	0.703	(Martini *et al.* 2006)
WT	0.014	0.031	2.176	0.958	0.956	(Keelagher *et al.* 2011)
*exo1*	0.064	0.357	5.609	0.604	0.602	(Keelagher *et al.* 2011)
WT	0.018	0.018	0.995	0.968	0.965	(Wanat *et al.* 2008)
*csm4*	0.053	0.317	6.002	0.644	0.647	(Wanat *et al.* 2008)
*ndj1*	0.058	0.208	3.615	0.749	0.746	(Wanat *et al.* 2008)
*msh5*	0.046	0.608	13.328	0.356	0.374	(Wanat *et al.* 2008)
*mlh1*	0.069	0.296	4.273	0.677	0.655	(Wanat *et al.* 2008)
*ndj1 csm4*	0.033	0.132	3.986	0.847	0.839	(Wanat *et al.* 2008)
*msh5 csm4*	0.095	0.706	7.463	0.217	0.266	(Wanat *et al.* 2008)
*mlh1 csm4*	0.154	0.507	3.302	0.418	0.417	(Wanat *et al.* 2008)
WT	0.081	0.017	0.209	0.911	0.904	(Ghosh *et al.* 2004)
*iml3*	0.372	0.203	0.547	0.502	0.500	(Ghosh *et al.* 2004)
WT	0.030	0.024	0.792	0.950	0.946	(Masison and Baker 1992)
*cbf1*	0.175	0.137	0.783	0.734	0.713	(Masison and Baker 1992)
WT	0.013	0.010	0.767	0.980	0.978	(Jessop *et al.* 2006)
*sgs1*	0.069	0.036	0.528	0.987	0.897	(Jessop *et al.* 2006)
WT	0.008	0.007	0.871	0.898	0.985	(Jessop *et al.* 2006)
*sgs1*Δ*795*	0.134	0.054	0.404	0.822	0.819	(Jessop *et al.* 2006)

The best-fit value for random spore death (RSD) and nondisjunction death (NDD) generated by TetFit are shown along with the ratio of RSD and NDD. The observed spore viabilities (Obs SV) and calculated best fit spore viabilities (Cal SV) are also given.

**Figure 7:**
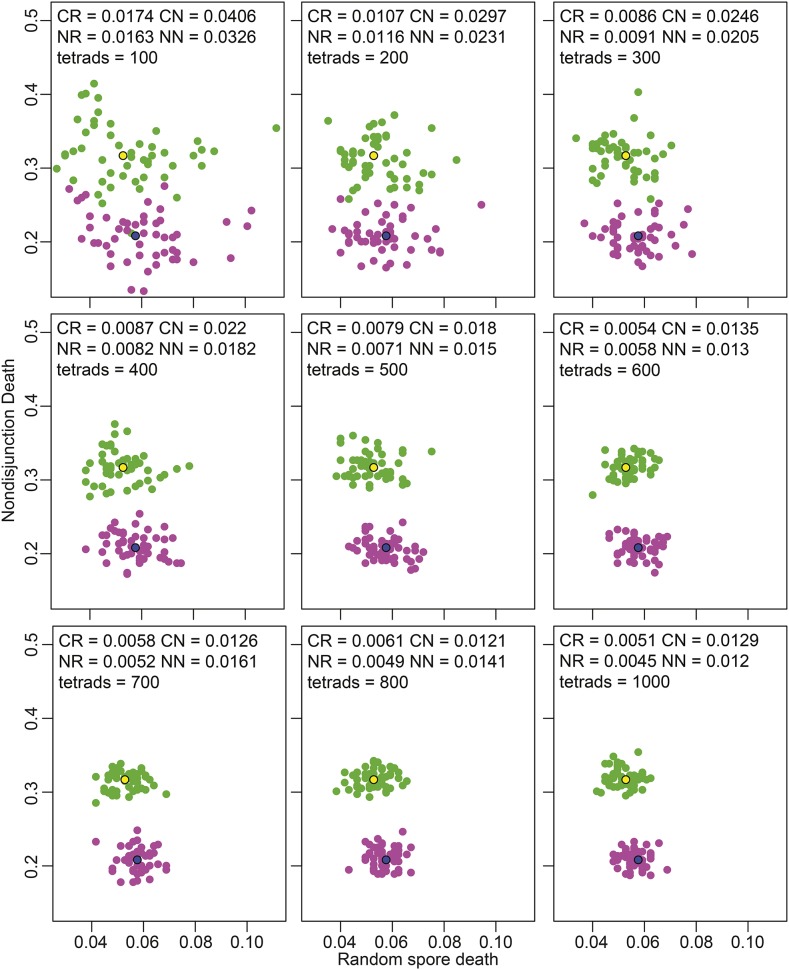
Analysis of variability from a varying number of tetrads analyzed. Simulations were performed using 100–1000 simulated dissected tetrads using the best fit RSD and NDD for *csm4*Δ and *ndj1*Δ from the Wanat *et al.* (2008) dataset. For each simulation 50 sets of tetrad dissections were simulated for *csm4*Δ and *ndj1*Δ. Best fits for *csm4*Δ and *ndj1*Δ are shown with black borders in yellow and blue, respectively. Simulations for *csm4*Δ and *ndj1*Δ are shown in green and purple, respectively. The standard deviations of each simulation for *csm4*Δ RSD (CR), *csm4*Δ NDD (CN), *ndj1*Δ RSD (NR), and *ndj1*Δ NDD (NN) are given.

We next tested the robustness of TetFit by running simulations based on the calculated NDD and RSD values determined for *csm4*Δ and *ndj1*Δ using different numbers of tetrads (Figure 7). For these two mutants, with fairly similar defects in spore viability, we found that 200 tetrads is a reasonable number to distinguish their effects on NDD and RSD.

**Figure 8:**
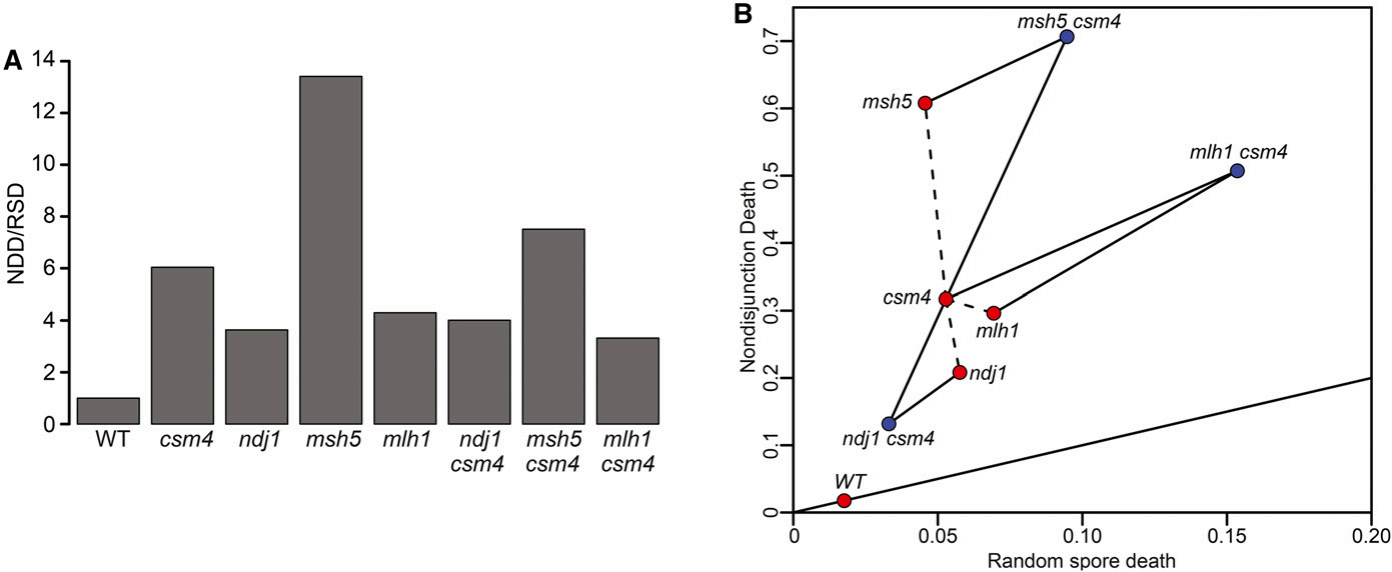
Genetic analysis of double mutants. (A) Bar plot of the NDD/RSD ratios from the Wanat *et al.* (2008) data set. (B) Scatter plot of RSD and NDD using the Wanat *et al.* (2008) data set. Single mutants and WT are shown in red and double mutants are shown in blue. Solid lines connect the double mutants to their respective single mutants, and dashed lines connect the single mutants together. A solid line with a NDD/RSD ratio of 1 is shown.

The ability to separate the relative contributions of RSD and NDD is also a useful tool for genetic epistasis analysis. Both the *csm4*Δ *msh5*Δ and *csm4*Δ *mlh1*Δ double mutants give increased levels of RSD and NDD compared to their respective single mutants (Figure 8A). The NDD/RSD ratio for *csm4*Δ *msh5*Δ is intermediate to those for each single mutant, suggesting that *csm4*Δ and *msh5*Δ both increase NDD and RSD through independent pathways.

By contrast, the NDD/RSD ratio for *csm4*Δ *mlh1*Δ is lower than for each single mutant. By comparing the absolute NDD and RSD values, it appears that *csm4*Δ *mlh1*Δ exhibits less NDD and more RSD than expected from the single-mutant phenotypes, suggesting a more complex relationship between the two mutations than previously observed (Figure 8B; Wanat *et al.* 2008). That is, the two mutations act together to reduce NDD, perhaps at the expense of increased RSD.

Both RSD and NDD are reduced in the *ndj1*Δ *csm4*Δ double mutant compared to each single mutant (Figure 8B). The *ndj1*Δ *csm4*Δ double mutant has been shown previously to interact genetically in a partial-reciprocal epistastic manner, in which the phenotype of the double mutant is weaker than either of the single mutants (Wanat *et al.* 2008). Our results are consistent with such a relationship since it appears that is that *csm4*Δ suppresses the increased RSD phenotype of the *ndj1*Δ mutant (presumably due to PSCS), and that *ndj1*Δ suppresses the NDD phenotype of *csm4*Δ.

## Discussion

Here, we describe a computational approach (TetFit) to estimate the contributions of meiosis I nondisjunction (MI-ND) and random spore death (RSD) to spore viability in WT and mutant strains. We show that low levels of spore inviability observed for WT strains can be explained by a combination of both RSD and MI-ND, where RSD may be due to precocious sister chromatid separation or meiosis II nondisjunction events. We also find combinations of MI-ND and RSD that can account for spore inviability in a battery of mutant strains defective for meiotic chromosome events. In addition, TetFit can uncover additional contributions to spore death not accounted for by MI-ND or RSD. Thus, TetFit is an effective tool for yeast researchers to uncover the molecular basis for spore inviability in uncharacterized strains.

TetFit will also calculate the average frequency of MI-ND per chromosome (Avg-ND), which has some advantages to measuring MI-ND for individual chromosomes. To date, no comprehensive analysis of the MI-ND frequency of all 16 chromosomes of budding yeast has been performed in either WT or mutant strains. As such, the full range of MI-ND frequencies for each chromosome is not known. Shonn *et al.* (2000) examined four chromosomes of differing lengths and found no size dependence on MI-ND in WT cells, but increasing levels of MI-ND for longer chromosomes in a *mad2*Δ background (Table 1). For most strains analyzed, MI-ND frequencies of only one to three chromosomes were determined (Table 1). Even so, MI-ND frequencies for the same chromosome can vary across platforms (Table 1). For example, the MI-ND frequency of chromosome III in the *csm4*Δ mutant has been reported in three different publications, and ranges from 1.9%, 7.8%, to 12% (Table 1). Analysis of artificial chromosome constructs has shown that shorter chromosomes are more likely to missegregate during mitosis than longer chromosomes (Murray *et al.* 1986). Nonetheless, it is not possible, *a priori*, to predict a given mutation may or may not exhibit chromosome-specific effects. The method described here has an increased chance of detecting an MI-ND phenotype.

In mutant backgrounds with a moderate level of MI-ND, analyzing MI-ND frequencies through dominant selectable markers can require dissecting a very large number of tetrads, far more than needed to generate reliable genetic maps. For example, Wanat *et al.* (2008) dissected 1164 and 1400 tetrads of *csm4*Δ and *mlh1*Δ mutants, respectively, and compared their MI-ND frequency to WT (1199 tetrads). While these numbers were sufficient for measuring map distances, only three and five Ch XV MI-ND events were detected for *csm4*Δ and *mlh1*Δ, respectively (Wanat *et al.* 2008). A Fisher’s exact test comparing WT to *csm4*Δ and *mlh1*Δ, assuming WT had no MI-ND events, gives *P* values of 0.12 and 0.13 (not adjusted for multiple comparisons), respectively. In these cases, loss of Csm4 or Mlh1 does not appear to significantly increase MI-ND. For strains with lower levels of MI-ND, the work required to detect increases in MI-ND frequencies would be prohibitive.

Using TetFit described here requires the analysis of as few as 200 dissected tetrads to generate RSD and MI-ND data for analysis. The low numbers of tetrads required for each strain also facilitates epistasis analysis. Here we show proof-of-principle that phenotypes of single and double mutant strains can be quantitatively compared. Our analysis also provides additional insight into the nature of spore death that occurs in the double mutants *vs.* single mutants. We have also allowed for variable chromosome complements so that the TetFit can be easily customized for species other than *S. cerevisiae*. Importantly, the application of the R-Scripts does not require any special strain construction, and can be applied to previously observed tetrad distributions.

## Supplementary Material

Supporting Information
