# Supplementary material for: A Computational Approach to Estimating Nondisjunction Frequency in Saccharomyces cerevisiae
Source: G3 (Bethesda). 2016 Jan 8;6(3):669–82. doi: 10.1534/g3.115.024380 (PMC4777129; doi:10.1534/g3.115.024380)
Supplement: Supporting Information [file supp_6_3_669__index.html]

A Computational Approach to Estimating Nondisjunction Frequency in Saccharomyces cerevisiae — Supporting Information 

# A Computational Approach to Estimating Nondisjunction Frequency in *Saccharomyces cerevisiae*

## Supporting Information for Chu and Burgess, 2016

**Files in this Data Supplement:**

- Figure S1 - Effect of sporulation efficiency on tetrad distributions. (.pdf, 381 KB)
- File S1 - R-Script TetSim. TetSim is an R-script that simulates the expected distribution of tetrads giving 4, 3, 2, 1, or 0 viable spores due to random spore death. (.zip, 3 KB)
- File S2 - R-Script TetFit. TetFit is an R-Script that generates live:dead tetrad distributions and then determines which tetrad distribution best fits observed tetrad distributions. TetFit-A generates live:dead tetrad distributions from a user set range of RSD and MI-ND. TetFit-B generates live:dead tetrad distributions from a user set range of RSD and a fixed MI-ND. (.zip, 4 KB)
