# Supplementary material for: A Computational Approach to Estimating Nondisjunction Frequency in Saccharomyces cerevisiae
Source: G3 (Bethesda). 2016 Jan 8;6(3):669–82. doi: 10.1534/g3.115.024380 (PMC4777129; doi:10.1534/g3.115.024380)
Supplement: Supporting Information [file supp_g3.115.024380_FigureS1.pdf]

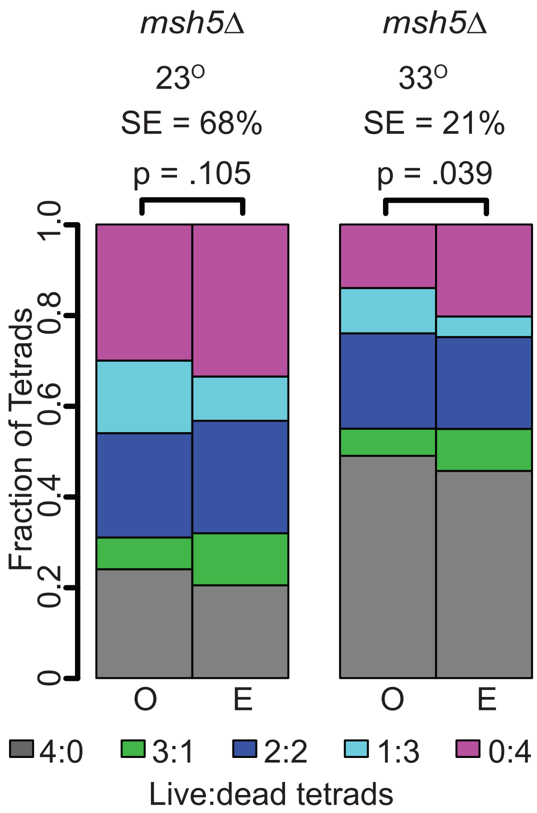

Figure S1. Effect of sporulation efficiency on tetrad distributions. Best fit tetrad distributions for the *msh5Δ* mutant at 23° (right) and 33° (left). The sporulation efficiency (SE) at 23° and 33° were 68% and 21% respectively.
